# Surgery for pain

**DOI:** 10.3171/2020.7.FOCVID2048

**Published:** 2020-10-01

**Authors:** Jason M. Schwalb, Kim J. Burchiel, Parag G. Patil, Tipu Z. Aziz

**Affiliations:** 1 Department of Neurosurgery and Center for Health Policy and Health Services Research, Henry Ford Health System, Detroit, Michigan;; 2 Department of Neurological Surgery, Oregon Health & Science University, Portland, Oregon;; 3 Departments of Neurological Surgery, Neurology, Anesthesiology, and Biomedical Engineering, University of Michigan, Ann Arbor, Michigan; and; 4 Nuffield Department of Clinical Neurosciences, University of Oxford, United Kingdom

Relief of pain is one of the primary aims of medicine. Relief of pain leads to longer life and greater quality of life. However, with the recognition of the opioid crisis in the United States, the inadequacy and adverse effects of pharmacotherapy are clear. Neurosurgical techniques for the treatment of pain have a long and storied history and are gaining increased enthusiasm as an alternative to opioids and other medications. Unfortunately, these techniques have not always been a standard part of neurosurgical training. Many of these procedures and their operative nuances may be unfamiliar to many neurosurgeons.

We are happy to present this collection of high-quality videos of neurosurgical techniques in the treatment of pain to help preserve the knowledge and experience of leaders in the field, to introduce new techniques and surgical refinements, and to serve as an educational resource. This collection represents the breadth of techniques in the neurosurgical treatment of pain, be they microsurgical, percutaneous, ablative, neurophysiologic, and/or neuromodulatory. We are hopeful that these videos, many from pioneers in the field, will be an enduring resource to surgeons wishing to add these procedures to their armamentarium or to further advance their existing techniques.

